# Microstructural Characterization of Calcite-Based Powder Materials Prepared by Planetary Ball Milling

**DOI:** 10.3390/ma6083361

**Published:** 2013-08-07

**Authors:** Wen-Tien Tsai

**Affiliations:** Graduate Institute of Bioresources, National Pingtung University of Science and Technology, Pingtung 912, Taiwan; E-Mail: wttsai@mail.npust.edu.tw; Tel.: +886-8-7703202; Fax: +886-8-7740134

**Keywords:** oyster shell, ball milling, powder material, surface characterization, true density

## Abstract

In this work, a planetary ball milling was used to modify the surface properties of calcite-based material from waste oyster shell under the rotational speed of 200–600 rpm, grinding time of 5–180 min and sample mass of 1–10 g. The milling significantly changed the microstructural properties of the calcite-based minerals (*i.e.*, surface area, pore volume, true density, and porosity). The surface characterization of the resulting powder should be macroporous and/or nonporous based on the nitrogen adsorption/desorption isotherms. Under the optimal conditions at the rotational speed of 400 rpm, grinding time of 30 min and sample mass of 5 g, the resulting calcite-based powder had larger specific surface area (*i.e.*, 10.64 m^2^·g^−1^) than the starting material (*i.e.*, 4.05 m^2^·g^−1^). This finding was also consistent with the measurement of laser-diffraction (*i.e.*, 9.7 *vs.* 15.0 μm of mean diameter). In addition, the results from the scanning electron microscope (SEM) observation indicated that surface roughness can be enhanced as particle size decreases as a result of particle-particle attrition. Thus, grinding the aquacultural bioresource by a high-energy ball milling can create the fine materials, which may be applied in the fields of inorganic minerals like aggregate and construction material.

## 1. Introduction

It is well known that the oyster shell is a natural biomaterial with excellent fracture strength and hard toughness, which are attributed to its laminated microstructure [1]. This mineral material is primarily composed of calcium carbonate (CaCO_3_) crystals (*i.e.*, calcite) laid down in a protein matrix [2,3]. To mitigate the environmental loads from waste oyster shell, many studies on its recycling or utilization have been reported in recent years. One of the methods was to reuse it as an available material without further thermal and chemical treatment such as desulfurization sorbent [4], sludge conditioner [5], adsorbent [6,7], construction material [8,9], nutrition supplement [10], eutrophication control medium [11,12], food additive [13], liming material [14], and dechlorinating agent [15].

Concerning the efficient utilization of waste oyster shell, the size reduction method could be used to fabricate finer powder because the surface properties (e.g., specific surface area) of powder material mainly depend on its particle size, particle shape, and pore structure [16]. The fine milling of granular material can not only reduce particle size but also change surface structure because of the mechanochemical effect [17,18]. Ball milling is a commonly used method of producing fine powder in many industries. The tumbling mill with centrifugal and planetary action has recently been used to prepare fine powder from a variety of materials such as minerals, ores, alloys, chemicals, glass, ceramics, and plant materials. More noticeably, the planetary ball milling can reduce particles to fine powders based on a mechanical energy transfer, or impact and friction forces through high hardness ball media [19,20]. However, its energy efficiency is low, and the power cost is high.

In the open literature, numerous prior studies have reported similar work on ball milling of other types of powders, but there is no report about the pore and surface properties of calcite-based mineral powder using planetary ball milling. In the present study, the mechanical grinding was used to process the local waste oyster shell in order to serve as a biomaterial powder. As demonstrated by the previous study [21], the ground eggshell powders, obtained by the planetary ball milling with varying rotational speed, grinding time and sample mass loading, have been analyzed to characterize their pore properties, particle sizes, surface morphologies, and crystallinities by N_2_ adsorption/desorption isotherm, laser diffraction, scanning electron microscope (SEM) observation, and X-ray diffraction, respectively. It was found that eggshell shells can be successfully ground to the resulting fine powder with the specific surface area of over 10 m^2^·g^−1^ while the specific surface area of its starting sample is less than 1.0 m^2^·g^−1^. Thus, the main objective of this work was to explore the preparation of fine minerals from waste oyster shells using a high-energy ball milling as a function of rotational speed, grinding time, and mass dosage, and to characterize the surface properties of the resulting powders.

## 2. Experimental Section

### 2.1. Materials

**Figure 1:**
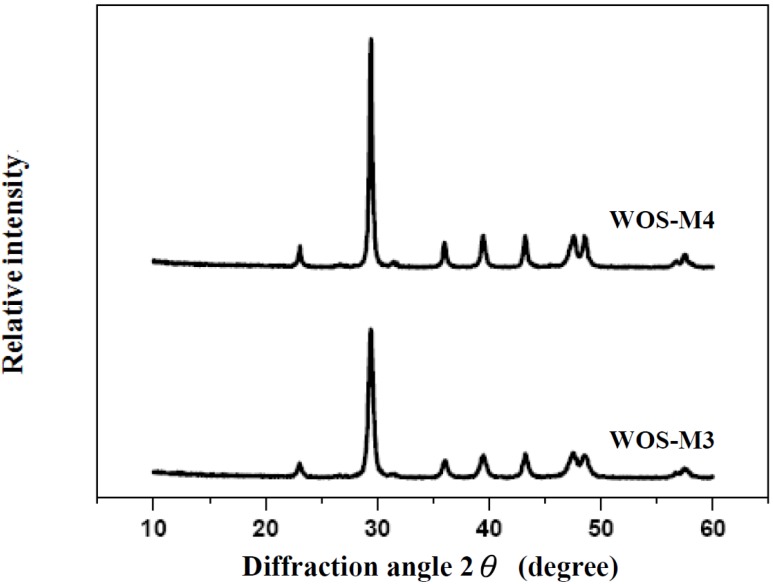
X-ray diffraction (XRD) diffractograms of some milling shell powders (*i.e.*, WOS-M3 and WOS-M4).

The waste oyster shell (denoted as WOS) was obtained from giant Pacific oyster (*Crassostrea gigas*) in a commercial market (Kaohsiung City, Taiwan). The shell sample was first cleaned with tap water to remove fresh remnants attached to the shells, and then it was dried by the solar radiation for at least 2 h. The shells were further crushed and ground by a rotary knife cutter to prepare smaller particles. The particle materials were thus sieved to the mesh number ranges from 100 to 200 (average particle size = 0.112 mm) according to the standard sieve designation. The resulting materials were finally dried at 105 °C for 24 h and stored in the desiccator prior to the physical and chemical characterizations. The crude shell particle (denoted as WOS-RM) was used as raw material further ground to fine powder by planetary ball milling described below. As listed in Table 1, an inductively coupled plasma-atomic emission spectrometer (Jarrel-Ash Co., USA; Model No.: ICAP 9000) and an elemental analyzer (Model: vario EL III; Elementar Co., Germany) have been used to obtain the chemical compositions of the WOS-RM sample, showing that the sample is mainly composed of calcium carbonate (*i.e.*, calcite). On the other hand, the X-ray diffraction (XRD) pattern of the sample using a Rigaku MiniFlex instrument (Cu-Kα radiation) exhibited a significant peak of around 30 degree (2*θ*), which is characteristic of crystalline calcite that indicates as *hkl* (104) [22]. As seen in Figure 1, the diffraction peaks (not shown here) were broadened as the ball milling (described below) increased, indicating a decreasing trend in crystallite (grain) sizes due to the internal stress [19]. Based on the Scherrer equation, the crystallite size is reversely proportional to the width at half maximum of the broadened diffraction line on the 2*θ* scale. Accordingly, the crystallite size of WOS-M3 should be less than that of WOS-M4. The decrease of the calcite crystal size may be attributed to the thermal expansion while high-impact grinding results in the deformation.

**Table 1:** Chemical composition of waste oyster shell used as raw material in this work.

Inorganic element (wt %) ^a^									Organic element (wt %) ^b^		
Ca	Mg	Si	Al	Fe	Sr	Na	P	Ti	C	H	N
35.3	0.388	0.557	0.431	0.223	0.09	1.06	0.08	0.013	11.55	0.48	0.00

^a^ Analyzed by the inductively coupled plasma-atomic emission spectrometer; ^b^ Analyzed by the elemental analyzer.

### 2.2. Experimental Apparatus and Procedures

In the present study, a dry process by using high hardness material as grinding medium was used to make fine calcite-based particle. The fine grinding of crude shell sample was performed by a tumbling (planetary) ball mill (Retsch Co., Germany; Model No.: PM 100). The apparatus was equipped with a pair of agate jars. The inner diameter and depth in the 50 cm^3^-cylindrical pot were 60 mm and *ca.* 30 mm, respectively. The polished zirconium oxide ball with 20 mm diameter laid inside the jar was used to grind the sample. For each grinding experiment, the sample was put into the milling pot and ground under the following operation modes:

1. Rotational speed: 200, 300, 400, 500, and 600 rpm (denoted as S1, S2, S3, S4, and S5, respectively);
2. Grinding time: 5, 10, 30, 60, 120, and 180 min (denoted as T1, T2, T3, T4, T5, and T6, respectively);
3. Sample mass: 1.0, 2.5, 5.0, 7.5, and 10.0 g (denoted as M1, M2, M3, M4, and M5, respectively).

After being ground for the prescribed operation mode, the ground powder product was thoroughly removed from the mill jar and then stored in a desiccator for the subsequent characterization measurements. The preparation conditions and sample identification codes of the resulting oyster shell powders were denoted as WOS-S series (the preparation under the prescribed conditions: sample mass of 5 g, rotational time of 30 min, and clockwise rotation), WOS-T series (the preparation under the prescribed conditions: sample mass of 5 g, rotational speed of 400 rpm, and clockwise rotation), and WOS-M series (the preparation under the prescribed conditions: rotational time of 30 min, rotational speed of 400 rpm, and clockwise rotation). For example, the shell powder WOS-S1 was prepared under the grinding conditions of rotational speed of 200 rpm, sample mass of 5 g, and rotational time of 30 min.

### 2.3. Characterization of Resulting Powder Materials

#### 2.3.1. Pore Property

Regarded as a comparative factor in determining the degree of pore properties of the resulting powders in the present study, the Brunauer-Emmett-Teller (BET) surface area has generally been used for comparing the specific surface areas of a variety of porous materials. The pore properties of fine powder, including the BET surface area (S_BET_, m^2^·g^−1^) and pore size distribution, were obtained while its nitrogen adsorption-desorption isotherms were measured at −196 °C. The Surface Area & Porosity Analyzer (Micromeritics Co., Norcross, GA, USA, Model No.: ASAP 2020) was used to examine this work. The pore size distribution was calculated based on differential pore volume of Barrett-Joyner-Halenda (BJH) desorption isotherm using the Kelvin model of pore filling theory [16].

#### 2.3.2. True Density

In the measurement of true density, the contribution to the volume of pores or internal voids must be subtracted based on its definition. Helium gas can penetrate even the very small pores in the powder sample because of its inertness and small molecule size (about 0.2 nm). A helium displacement method with a pycnometer (Micromeritics Co., Norcross, GA, USA, Model No.: AccuPyc 1340) was used to measure the true density (ρ_s_) of the particulate sample in this work. From the data on total pore volume and true density of the fine particle, its porosity can be thus estimated [16].

#### 2.3.3. Particle Size Distribution

In addition to the pore property, the particle size also contributes to the surface area of the resulting fine powder. An LS 230 (Micro-volume module) laser-diffraction particle size analyzer (Beckman Coulter Inc.; USA) was used to examine the particle size distributions of crude shell particle (*i.e.*, WOS-RM) and some resulting fine powders. Prior to the measurement, about 0.1 g was added to 30 cm^3^ de-ionized water to prepare a suspension solution. Then, magnetic agitation was used to shake the suspension solution to avoid the sedimentation of the sample particle.

#### 2.3.4. Scanning Electron Microscope (SEM) Observation

In order to elucidate the particle properties (e.g., surface roughness, pore property and particle size) of the resulting shell powders from nitrogen adsorption/desorption isotherms and laser diffraction, the scanning electron microscope (SEM) was used to further examine and observe the external texture. The surface morphologies of the resulting fine powders were examined using the scanning electron microscopy (SEM) by a S-3000N (Hitachi Co., Tokyo, Japan) apparatus operated at a 15.0 kV accelerating potential. Prior to the observation, the surface of the sample was coated with a thin, electric conductive gold film.

## 3. Results and Discussion

### 3.1. Pore Property

**Figure 2:**
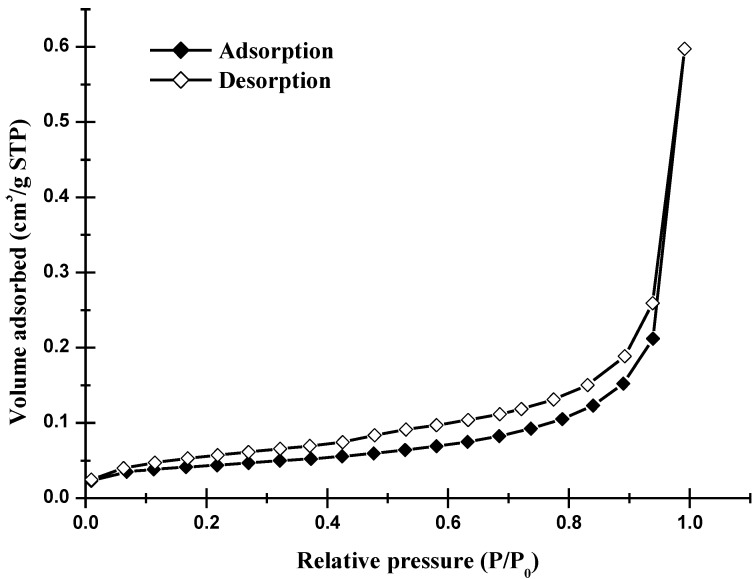
N_2_ adsorption-desorption isotherms of the optimal calcite-based powder (*i.e.*, WOS-M3).

**Figure 3:**
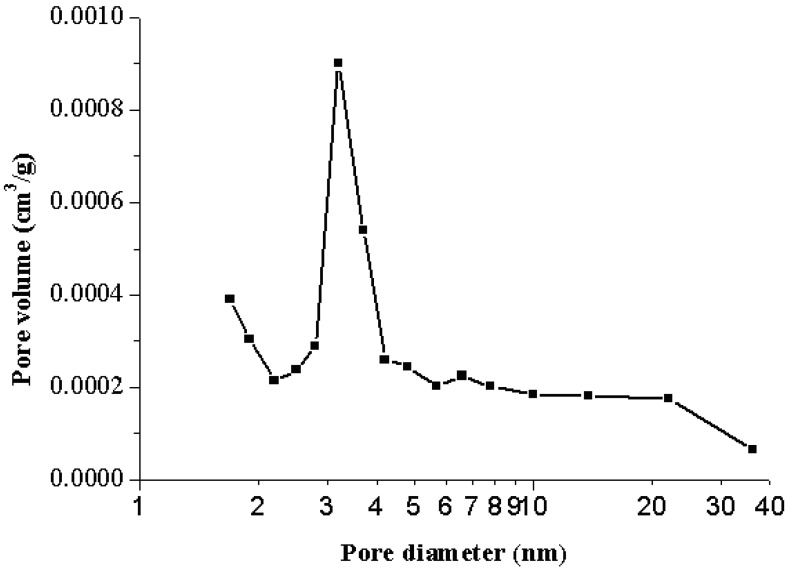
Pore size distribution of the optimal calcite-based powder (*i.e.*, WOS-M3).

The data in Table 2 indicated the Brunauer-Emmett-Teller (BET) surface area, total pore volume, true density of the starting sample (*i.e.*, WOS-RM), and the resulting WOS-series. The data revealed that most of these fine minerals possessed poorer pore properties toward the probe molecule (*i.e.*, nitrogen) than common porous materials such as activated clay [23]. Figure 2 showed the nitrogen adsorption/desorption isotherms of the optimal calcite-based powder (*i.e.*, WOS-M3), indicating that monolayer-multilayer adsorption is unrestricted. On the basis of the Brunauer, Deming, Deming, and Teller (BDDT) classification [24], the isotherms belong to typical Type II, which has characteristic that nonporous or macroporous solids where monolayer coverage is succeeded by multilayer adsorption at higher relative pressure values. However, it should be noted that a small hysteresis loop can be seen in Figure 2. The probe molecule gas (*i.e.*, nitrogen) was progressively added to the system to measure the lower (adsorption) branch of the loop while the probe molecule gas was progressively withdrawn from the system to measure the upper (desorption) branch. According to the classification of isotherm shapes, the Type VI isotherms possess a hysteresis loop, which is associated with mesoporous solids, where capillary condensation occurs [24]. Furthermore, the hysteresis loop, which is corresponding to type H3 recommended by the International Union of Pure and Applied Chemistry (IUPAC), has been associated with solids having slit-shaped pores with wide mouths [24]. From the pore size distribution (<50 nm) of WOS-M3 powder (Figure 3) based on the pore volumes of the Barrett-Joyner-Halenda (BJH) desorption branch in the measurement of nitrogen isotherms, it was observed that the pores of the optimal mineral sample have a heterogeneous distribution of slit widths mainly ranging from 2.5 nm to 4.5 nm. The foregoing observation was also consistent with the N_2_ adsorption/desorption isotherms as seen in Figure 2.

**Table 2:** Physical characterization of the crude oyster shell powder (denoted as WOS-RW) and the calcite-based mineral powders (denoted as WOS-series).

Sample ID	BET surface area (m^2^·g^−1^)	Total pore volume (cm^3^·g^−1^)	True density (g·cm^−3^)	Porosity (-) ^f^
WOS-RW	4.05	0.0244	2.596	0.06
WOS-S1 ^a^	5.52	0.0276	2.622	0.07
WOS-S2 ^a^	7.31	0.0532	2.611	0.12
WOS-S3 ^a,d^	10.64 ^e^	0.0660	2.606	0.15
WOS-S4 ^a^	9.18	0.0579	2.584	0.13
WOS-S5 ^a^	8.04	0.0454	2.575	0.11
WOS-T1 ^b^	4.14	0.0359	2.620	0.09
WOS-T2 ^b^	7.37	0.0429	2.621	0.10
WOS-T3 ^b,d^	10.64 ^e^	0.0660	2.606	0.15
WOS-T4 ^b^	9.20	0.0682	2.590	0.15
WOS-T5 ^b^	6.30	0.0382	2.599	0.09
WOS-T6 ^b^	5.92	0.0322	2.582	0.08
WOS-M1 ^c^	5.41	0.0484	2.508	0.11
WOS-M2 ^c^	7.20	0.0536	2.577	0.12
WOS-M3 ^c,d^	10.64 ^e^	0.0660	2.606	0.15
WOS-M4 ^c^	7.06	0.0474	2.636	0.11
WOS-M5 ^c^	6.11	0.0368	2.641	0.09

^a^ The grinding operation was set as a function of rotational speed (200, 300, 400, 500, and 600 rpm; denoted as S1, S2, S3, S4, and S5, respectively) under the prescribed conditions: sample mass of 5 g, rotational time of 30 min, and clockwise rotational direction; ^b^ The grinding operation was set as a function of grinding time (5, 10, 30, 60, 120, and 180 min; denoted as T1, T2, T3, T4, T5, and T6, respectively) under the prescribed conditions: sample mass of 5 g, rotational speed of 400 rpm, and clockwise rotational direction; ^c^ The grinding operation was set as a function of sample mass (1.0, 2.5, 5.0, 7.5, and 10.0 g; denoted as M1, M2, M3, M4, and M5, respectively) under the prescribed conditions: rotational time of 30 min, rotational speed of 400 rpm, and clockwise rotational direction; ^d^ WOS-S3, WOS-T3 and WOS-M3 are identical; ^e^ 10.64 ± 0.25 m ^2^ g ^−1^ (N = 3); ^f^ The porosity of particle was estimated by its total pore volume and true density [16].

In the rotation-ground shell particle series (WOS-S series), the crack or slit seemed to be created by rotation speed of increasing from 200 to 600 rpm. As a result, the pore properties of BET surface area increased from 5.52 m^2^·g^−1^ at rotational speed of 200 rpm to 10.64 m^2^·g^−1^ at rotational speed of 400 rpm. Moreover, the pore property of the resulting powder increased to the maximum at about 400 rpm, but steadily declined in rotational speed at above 400 rpm. The optimum value could result from a compromise among the stress imposed to each particle, the mobility and attrition resistance of the sample particles, and the collision frequency and intensity. This result was in high accordance with the previous observation on the eggshell ground by a planetary ball mill [23].

As listed in Table 2, when the grinding time increased from 5 min to 30 min, the pore properties of the WOS-T samples were also increased as a result of the development of mesopores (crack or slit) and/or fine powder. However, it was observed that the values at grinding time of 30–60 min decreased more slightly than those at grinding time less than 30 min possibly due to the breakdown of the few well developed pores when the grinding time was longer than the crack transition time.

Table 2 showed the pore properties of the WOS-M samples in terms of BET surface area produced at a rotational speed of 400 rpm and a milling time of 30 min under various sample mass dosages (1.0–10.0 g). Clearly, the BET surface areas of the resulting powder solids tended to gradually increase with the increase in the mass dosage from 1.0 to 5.0 g. However, there was an optimum value of BET surface area at the minimum mass dosage (*i.e.*, 5.0 g) for the sample WOS-M3 because of the extent of the crack or slit evolution and development of fine particle. As the grinding was operated at mass dosage of above 5.0 g, a more inefficient particle-particle contacts and a lower energy transmitted to each particle contributed to the decrease of the pore property. As a result, the optimum operation parameters may result from a compromise between the stresses imposed on each particle, the mobility and attrition resistance of the sample particles, and the collision frequency and intensity.

### 3.2. Particle Size Distribution

**Figure 4:**
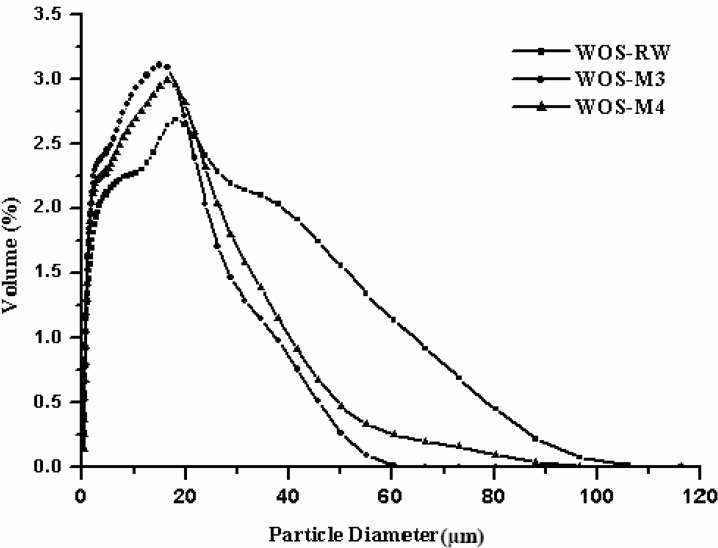
Particle size distributions of the crude oyster shell powder (denoted as WOS-RW) and some calcite-based powders (*i.e.*, WOS-M3 and WOS-M4).

Figure 4 depicted the evolution in the size distributions of the crude shell particle (*i.e.*, WOS-RW) and two resulting particles (*i.e.*, WOS-M3 and WOS-M4), which are representative of fine bioceramics with a definite division of pore properties as listed in Table 2. The particle size distribution of the WOS-RW sample broadly ranges from 0.4 to 120 μm, but it reaches a peak at about 20 μm. Approximately 90% of the crude sample particles possess particle size below 40 μm. When the size reduction evolved during grinding, the pattern mode of the particle size distribution and its corresponding peak are shifted from the right hand to the left hand. This led to a somewhat reduction of the mean diameter from 15.0 μm of the crude sample (*i.e.*, WOS-RW) to 9.7 μm of the WOS-M3 sample. However, the median size of the WOS-M4 sample was increased to 10.8 μm. This should result from the agglomeration, which may occur according to a coalescence mechanism when the mass loading of the oyster shell sample is too high. Generally, the fragmentation rate decreases with an increase of the particle sample mass loading because inefficient particle-particle contacts are more important and the energy transmitted to each particle is lower [25]. If the oyster shell sample is assumed to be uniform with the spherical type in the particle size, its external surface area is reversely proportional to its particle diameter. Consequently, the order of the specific surface areas would be: WOS-M3 > WOS-M4 > WOS-RW. Obviously, the data on the particle size and its distribution were in accordance with the results of pore properties (seen in Table 2).

### 3.3. Scanning Electron Microscope (SEM) Observation

**Figure 5:**
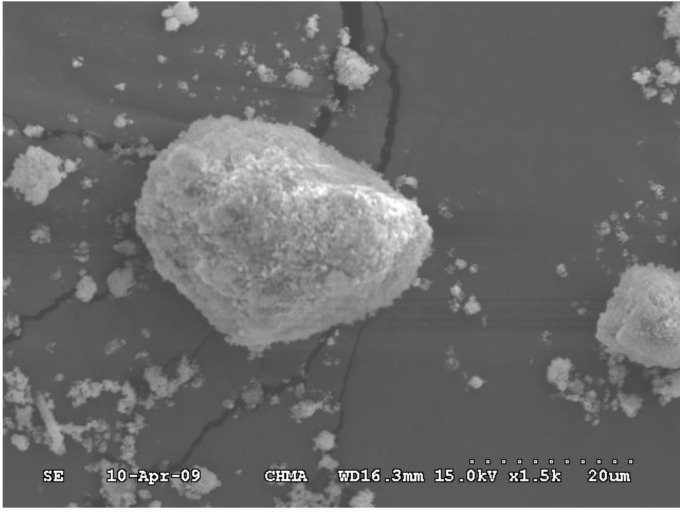
Scanning electron microscope (SEM) photograph (magnifications: 1500×) of the optimal calcite-based powder (WOS-M3).

The textural structure examination of the optimal calcite-based sample (*i.e.*, WOS-M3) was further observed from the SEM photograph to elucidate the grinding effect on the particle size. From Figure 5, it can be clearly seen that the ground shell sample and its agglomerate had a much smaller particle size. It seems that the sample does not possess well-defined pore structures. The finding was in agreement with the results of N_2_ adsorption-desorption isotherms (Figure 2). However, the powder particle displayed a much rougher and irregular surface structure by the mechanical mechanism during grinding, leading to the formation of some pores/cracks on the surface. This surface roughness and macropores can also exhibit the apparently larger BET surface areas of the resulting shell samples as described above (Table 2).

## 4. Conclusions

In conclusion, planetary ball milling method has been used to grind the oyster shell waste under various conditions. In order to characterize their properties, N_2_ adsorption/desorption isotherms, laser diffraction, and SEM observation have been examined to analyze the resulting biomaterial powders. The following conclusions can be drawn:

- Under the rotational speed of 200–600 rpm, grinding time of 5–180 min and sample mass loading of 1–10 g, the grinding treatment significantly changes the surface properties of the calcite-based minerals. The pore properties of the optimal resulting powder are 10.64 m^2^·g^−1^, 0.066 cm^3^·g^−1^, and 0.15 based on BET surface area, total pore volume and porosity, respectively, as compared to those (*i.e.*, 4.05 m^2^·g^−1^, 0.024 cm^3^·g^−1^, and 0.06) of the starting material. This finding was also consistent with the particle size measurement (*i.e.*, 9.7 *vs.* 15.0 μm of mean diameter).
- From the nitrogen adsorption/desorption isotherms, a typical Type II was found in the resulting powders, indicating that the isotherms are characterized by the nonporous materials or materials with macropores or open voids. However, a small hysteresis loop was also seen in the isotherms, suggesting that a small amount of mesopore with wide slit-shaped mouth exists in the fine powder.
- According to the observations in the SEM, the surface roughness can be enhanced as particle size decreases as a result of particle-particle attrition, suggesting that the specific surface areas of resulting powders hence increase with increasing fractural impact during high-energy ball milling.
